# Resonant Retiring? Experiences of Resonance in the Transition to Retirement

**DOI:** 10.3389/fsoc.2021.723359

**Published:** 2021-10-26

**Authors:** Luisa Bischoff, Annette Franke, Anna Wanka

**Affiliations:** ^1^ Research Training Group “Doing Transitions”, Goethe University, Frankfurt, Germany; ^2^ Department of Social Work, Protestant University of Applied Sciences, Ludwigsburg, Germany

**Keywords:** resonance, transitions, retirement, world relationship, social inequality

## Abstract

In the process of life course transitions, relations between the self and the world transform, which can according to Hartmut Rosa be framed as resonance. This article focuses on the retirement transition and thus on the exit from gainful employment as one of the central spheres of our world relationship in late modernity. It raises the following questions: How do experiences of resonance change in the course of the retirement transition? Does the loss of gainful employment lead to disruptions or even the absence of resonance in terms of alienation? And which role do dimensions of social inequality, such as gender, income, education or mental health status play for resonance transformations in the transition to retirement? In terms of a reflexive mixed-methods design, this article combines quantitative panel data from the German Ageing Survey (2008–17) with a qualitative longitudinal study from the project “Doing Retiring” (2017–21). Our results show that the transition from work to retirement entails a specific “resonance choreography” that comprises a phase of disaffection (lack of resonance) at the end of one’s working life followed by a liminal phase in which people search for intensified experiences of resonance. We outline practices in which transitioning subjects seek out resonance, and the experiences they make within this process according to their social positions. We thereby find that the desire for resonance tends to be beyond intentional resonance management which manifests in products and services like coaching or wellness. In our conclusions, we discuss how resonance theory and retirement research/life course research can be fruitfully combined, but also highlight the methodological challenges the operationalization of resonance entails.

## Introduction

Retiring remains one of the major transitions within an individuals’ life course, and one that entails a variety of changes for the transiting individual: Income and occupational prestige may decrease, but so does work-related stress; similarly, work-related social networks may loosen, but private networks can be strengthened, and time is freed up for formerly neglected or new tasks and activities. The retirement transition is thus likely to initiate transformations of one’s relationships to the social world.

In his “Sociology of Our Relationship to the World” (2019), Hartmut Rosa characterizes these relations on a continuum between *resonance* (*Resonanz*) and *alienation* (*Entfremdung*). As a counter-term to alienation and a muted-unconnected interaction between world and subject, Rosa defines resonance as *a self-effective, uncontrollable mode of relation to the social, material, and transcendent world*: I allow myself to be touched and moved (by an encounter, a conversation, a nature experience etc.), I respond with my own voice (with an emotion, a bodily reaction like goosebumps), and within this process both I and the other one transform adaptively (through new insights, a new vividness).

Theoretically linking Rosa’s conception of resonance to the transition into retirement, we ask: Does the transition to retirement appear as a resonance-sensitive and -evoking life event? Does the parting from employment as one of the axes of resonance in modernity lead to the absence of resonance in terms of alienation? What potentials does this theory offer for retirement research in particular, aging research in general and vice versa? We firstly approach these questions theoretically by outlining the concept of resonance as well as linking it to notions of transitions and the life course. Secondly, we generate empirical research questions from our theoretical considerations, among others: Do specific resonance trajectories or “melodies” emerge across a transition process? How do chrononorms shape experiences of resonance in the transition process to retirement? Do experiences of resonance in the retirement transition vary based on social position? Following a mixed-method design, data from the German Ageing Survey (DEAS, 2008–17) are circularly contrasted with in-depth findings from three qualitative case studies from the longitudinal project “Doing Retiring” (2017–21, Goethe University Frankfurt, Germany). Finally, we perform a critical discussion of our empirical results in order to emphasize possible heuristic linkages between the study of retirement transitions and Rosa’s concept of resonance.

## Resonant Relations and Their Transformative Power

In his book “Resonance. A Sociology of Our Relationship to the World” (2019) Rosa aims to develop a “sociology of the good life”, for which he states that “the quality of a human life (and of social conditions) cannot be measured simply according to available options and resources (…) Whether a life is successful or unsuccessful depends on the ways in which world is or can be passively experienced and actively appropriated or adapted” (ibid., 26 ff.). He therefor goes beyond subjective theories of social identity, but rather places the relation of subject and world in the center, which is moderated by sociocultural conditions. At the same time, self-efficacy and empathy are crucial for individual perspectives on the world (as challenging or promising).^1^

According to Rosa (2013), modern Western societies are shaped by a new zeitgeist of “acceleration” (*Beschleunigung*)*,* e.g., of the pace of life (= increase and overlapping of actions and/or experiences per unit of time, as for example in multitasking). In addition, Rosa criticizes the hegemonic idea of modernity, namely the striving to expand the share of the world in terms of material goods, new technologies or life events (Baudrillard 1994). He especially highlights the paradox mechanism, that with seemingly fulfilling this striving – with every increase in time pressure, competition and mastery of the world –, the striving as well as the perspective on still unavailable parts grows and results in a loss of quality and disconnection in experiences (Rosa 2013). He describes the consequences of alienation as being social and individual issues at the same time such as the crisis of democracy, the environmental crisis and the psychological crisis (Rosa 2019). By interpretating depression and burn-out prevalence as individual effects of alienation, Rosa combines their occurrence with societal developments and prevents an individualized view on psychological phenomena (Ståhl 2020). Rosa criticizes the corrosive impact of acceleration toward *alienation* as a non-responsive, hostile relationship between subject and world.

He elaborates on Rahel Jaeggi’s (2016) extensive analyses of alienation and her notion of a “relation of relationlessness” (ibid., 3). According to her, the human being is not detached from the world in an encapsulated relationlessness way but is rather related to it in a deficient bond as “a stranger in the world that he himself has made” (ibid., 21). As a constructive solution to alienation and “world-muting” (*Weltverstummen*), Rosa sketches *resonance* as an essential component of human beings. As alienation as a concept seems to be well described in former social theory, he now aims to establish “resonance as a normative and descriptive metacriterion of successful life” (Rosa 2019, 451), focussing on how resonance can be developed.

**FIGURE 1:**
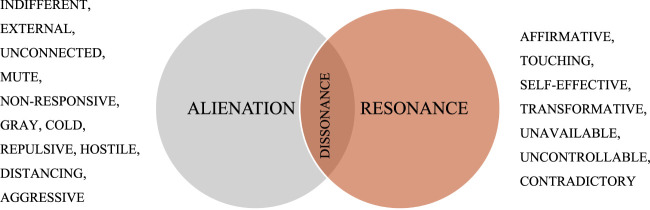
Rosa’s conception of alienation and resonance, own elaboration.

In Rosa’s understanding the relationship between subject and world follows a dialectical arrangement of attraction and repulsion, within which human beings are situated between alienation anxiety and resonance desire. This spectrum of alienation entails dimensions of indifferent disengagement, where “nothing speaks to me”, to a hostile-repulsive connection that appears aggressive toward me (Figure 1). Resonance as the counterpart represents a “vibrate wire” (*vibrierenden Draht*) between subject and the world, a momentary, processual emotional and affective experience of being overwhelmed, touched and transformed (Rosa 2019, 174) – eventually sensible in distinct ambiguous bodily experience like goosebumps or an increased pulse (Merleau-Ponty 1966). Furthermore, resonance implies more than just listening or echoing (this would be a “relation of relationlessness”), but rather emerges in a quadrature of *affecting* (“I let myself be touched by the Other”), *emotion* and *self-efficacy* (“We both actively respond with our own voices”), *mutual transformation* (“I and the Other are now no longer the same as before”) and *unavailability* or “accommodating” *uncontrollability* (“This moment cannot be planned, replicated or accumulated”) (Rosa 2020, 64).

Rosa conceptualizes resonance and alienation as co-constitutive: “resonance is only possible against the backdrop of a mute and unfamiliar Other, while, conversely, what is yet mute can only be affected or adaptively transformed on the basis of a prior or deep-seated, dispositional faith in resonance that feeds one’s hopes and expectations of being able to make some segment of world speak” (Rosa 2019, 190). Without the experience of lengthy, unpersonal conversations, one doesn’t feel the energetic impulse of a stimulating discussion. Using this example, we can describe Rosa’s distinction between resonance, consonance, and dissonance: Resonance is namely – as Rosa clearly emphasizes – not to be confused with pure harmony (ibid., 184). According to his argumentation, this consonance or echoing would not be a response with a voice of its own, but an indifferent relationship like a conversation with an exhaustive nod of the head, which makes a transformative adaption impossible. Then again, dissonance is not to be understood as a simple counterpart to resonance. Rosa rather distinguishes a form of “contradiction” (in our example: a heated, albeit respectful, debate) as possibly resonance-enhancing, while radical dissonance as a hurtful repulsion does not allow for resonance to occur and leads to alienation (ibid., 447).

Rosa’s idea of resonance goes beyond prominent sociological approaches focusing on subject-subject-interactions such as recognition (Honneth 1995) or collective excitement (Simmel 1976). Resonance represents a specific transformative mode of relation of the self to the world, as subject-subject relations, but also between human and non-human entities such as institutions, practices and objects (subject-object relations) (Latour 1996). Hence, Rosa describes the experiences of resonance on three durable resonance axes: horizontal (relationships with other human beings), diagonal (work, school, sports) and vertical (religion, art, music, nature). At the same time a guarantee or conscious production of resonance is – even within established resonance axes – hardly possible; the attempt is likely to be exposed as a reifying oasis or simulation of resonance (Rosa 2019, 186). However, with commercialized offers ranging from relaxing wellness experiences to adventurous extreme sports a market of resonance-promising “emodities”^2^ has been formed (Hochschild 1983; Illouz 2007). Explaining this impossibility of forcing resonance to emerge, Rosa distinguishes between adaptive world transformation processes (*Weltanverwandlung*) and the escalation principle of expanding reach or mastery of the world (*Weltaneignung*).

For Rosa, capacity or responsivity for resonance are basic human needs, which are however stimulated or dampened within existing social and cultural contexts (Rosa 2019). For example the current educational system does not only (re-)produce social inequalities, but also mediates our access to resonance axes: for the privileged, schools are places, where they find their own voice and experience self-efficacy, whereas schools appear to be zones of alienation (*Entfremdungszonen*) for the less privileged, as they are less likely to establish vertical axes of resonance (making music, visiting museums, protecting the environment) (ibid., 454). Additionally, Rosa frames resonance responsivity to be a mostly “female” phenomenon, since in Western culture the traits, that go along with it, like empathy, emotionality, or sensitivity, are associated with being “female.” But given that those cultures are being dominated by rationality and instrumentalization – mostly “male” traits – Rosa emphasizes, that resonance responsivity is being devalued, as are “female” traits (ibid.).

Despite a basic critical position toward escalation principles of capitalism Rosa does not describe his “sociology of the good life” as a political program but rather a “compass in contemporary political debates” (Rosa 2019, 458). He stresses the notion of resonance to question e.g., current standards of productivity (constant pressure, lack of self-efficacy) which are – considering the capitalist circumstances and a lack of basic income schemes – leading to increasing experiences of alienation (Lijster and Celikates 2019, 76). His critique is nevertheless understood to be an ethical – or even moralizing (Ståhl 2020) – critique of capitalism “and so, without an anchor in the present of class struggles, Rosa’s analysis accelerates aimlessly” (Blumenfeld 2018, 210). It is likewise questionable, if a resonant being in the world might not be enough to cope with the substantial and pervasive power of acceleration and alienation (Lijster and Celikates 2019). This critical stance goes even further, since in the sociological discourse the concept is being confronted with the accusation that it – by offering a Western, middle-class-based and romanticizing life agenda (Costa 2020; Simoulin 2020; Susan 2020) – disregards not only the positive elements of innovation but also existing gender inequalities or social relations of power and thereby current (im)possibilities to experience self-efficacy (Haubner 2017; Masquelier 2019; Susan 2020). Additionally, the inherent emphasis on simple, substantial-material activities of being resonance-enhancing (e.g., in the metaphor of baking bread) have been discussed as being open for neoliberal approaches (Haubner 2017; Beck 2019). Confronted with this criticism Rosa (2017) claims “on no point do I feel as misunderstood as on this one” (325). He instead emphasizes the idea of resonance to be the basis for an “affirmative revolution” (ibid., 311), a radical new category, which goes beyond previous demands for a new economic and social order with inherent elements of empowerment and social inclusion: “Resonance theory therefor aims to restore self-efficacy to the powerless” (Rosa 2019, 456). While some do appreciate Rosa’s optimistic perspective (Durou 2016; Masquelier 2019; Susan 2020), Micha Brumlik (2016) states, that this affirmative attitude, has abandoned the former irreconcilable but stimulating attitude of the Frankfurt School toward capitalism.

In light of this critique, the question arises as to how the descriptive-normative concept of resonance can be brought together with life course research and gerontological theories regarding retirement. In the following section, these approximations of the resonance concept to retirement research will be explored in more detail.

## Linking the Concept of Resonance to Retirement Transitions

Although Rosa (2020) already explicitly addresses individual transitions, he has not (yet) systematically connected his conception of resonance to transitions in the life course. To get a deeper understanding of possible heuristic links, we discuss resonance theory in terms of conceptions of transitions in general and the retirement process in particular. From our perspective resonance represents a momentary but also *processual relation* mode, which can be linked to concepts of transitions and taking pathways in the life course. As moments of uncertainty, transitions may enhance resonance but may also lead to anxiety for the passengers, when leaving the labor force entirely. According to Rosa (2020), certain life course transitions are particularly receptive or *porous* to experiences of resonance, such as childhood or puberty. Thus, also transitions in older age – like the transition into retirement – bear potential for experiences of resonance but also repulsive or hostile confrontation. The *dialectical architecture* of the subject-world-relationship in terms of alienation and resonance can be linked to the traditional approach of push and pull factors for retirement decision making. In addition, Rosa describes the sociocultural embeddedness of subject-world-relationships whereas retirement research often underlines *social policy frameworks and normative implications* for “adequate” timings of retirement. *Socioe*conomic aspects, like education, and mental health factors like self-efficacy in the resonance theory, are shaping the preconditions and resources of retirees before, during and after retirement. To explore possible fruitful connections between resonance theory and retirement research, we are focusing on the following dimensions:

- resonance and the *processual nature* of the retirement transition, which comprises a specific *porosity* and uncertainty about retirement as source of alienation or resonance
- resonance and *normativity* in the retirement transition process
- resonance and the implications of *social inequality* in the retirement transition process

### Resonance and the Processual Nature of the Retirement Transition

Anthropological transition research has long been concerned with how transitions are being structured as processes of status change. Arnold van Gennep (1909) formulated a chronological typology for transition processes: 1) Rites of separation (“Rites de Séparation”): individual’s detachment from their current state. 2) Transition rites (“Rites de Marge”): individual’s passing through a phase and 3) Rites of incorporation (“Rites d’Agrégation”): the durable position after a completed transition. Turner (1969) elaborated the phase of “transition rites” in his work as so-called “liminal phase” in which the individual can find itself to be ambiguous and disoriented. In this anti-structural “in-between world”, the threshold individuals find themselves in a relational constellation, which goes beyond their previous differences like social status or gender. These “communitas” as formulated by Turner emerge immediately, spontaneously, ambiguously, indeterminately, enhancing the power of structural change, just like resonance does. Although these communitas are not enduring, since their members will reintegrate into society again after the transition process, the liminal phase has a transformative impact, tying the participants to each other (ibid.).

Whereas such anthropological models claim to comprise all kinds of transitions equally, specific phase models have been developed to grasp the specificities of the retirement transition. A prominent example is Atchley’s (1971) phase model on retirement transition:

The first phase of “pre-retirement” starts about 3 years before the retirement with still vague ideas about exit the labor force. In this phase, the future time after retirement is perceived positively as a “remote phase”. The “honeymoon phase” starts immediately after one stops working and comprises the first weeks in retirement. Depending on individual health and life situation, retirees enjoy the free time and relief from workload. In the “disenchantment phase”, however, a short period of disillusion follows and first doubts arise about the legitimacy of retirement in everyday life. Depending on their resources, persons may become vulnerable for mental health impairments (e.g., depressions). Individuals then enter the next “phase of reorientation”, where retirees start to cope with the situation. According to Atchley, the fourth phase can last a relatively long time, depending on individual options and resources. A successful passing of the fourth phase leads over to the “phase of stability”. Here individuals are heading into the longest and most endurable period of retirement. They are aware of their opportunities and limitations in retirement and develop adequate routines and activities, which provide them enjoyment and self-affirmation. In the last “phase of termination” their health decreases significantly and their need of help and care increases. As a result, the role as retirees recedes in the background and daily routines are primally linked to impaired health status and frailty.

Obviously, Atchley’s phase model assumes a typical male working biography characterized by transitioning from traditional fulltime employment into retirement. However, in combination the models of van Gennep, Turner and Atchley point to retirement being a dynamic, ambiguous process framed by personal resources, which may entail resonance transformations. This transition requires awareness, reflexivity, evaluation and developing strategies to cope with new roles in order to obviate mental health concerns and social exclusion.

If we now combine this process ontology of retirement transitions with the theory of resonance, we can ask: How do experiences of resonance and alienation change during the process of transiting to retirement? Do specific resonance trajectories or “melodies” emerge empirically across a transition process?

### Resonance and Normativity in the Retirement Transition Process

Regarding the normativity of transitions in general, Andreas Walther (2020) refers to different constellations of discourses, norms, and normalities, as well as to the social institutions in which they manifest themselves, as *transitional regimes*. They are based on an underlying normative time structure that orchestrates the life course in respective regimes. With Elizabeth Freeman (2010) this structure can be referred to as *chrononormativity*, which describes in particular (but not exclusively) perceptions about the “right” time for specific life stages and life course transitions, such as going to school, starting a family, or retiring. These perceptions manifest and stabilize themselves in legal regulations and executive organizations, such as the age-determined school system, the legal right to marry at a certain age, or the statutory retirement age. Identifying one’s own position in this life course allows to ascribe biographical meaning to (life) events, to position oneself, and to construct identities. Despite an increasing de-standardization, such prepositions of an “adequate timing” in the life course hardly lose their individual and societal significance (Kohli 2007; Wanka 2020).

Specifically, in regard to retirement: Silke Van Dyk et al., 2013 traced significant changes in representations of a “good retirement life” in political and public media retirement discourses that took place in Germany (and most of the Western world) since the late 1980s. Their findings suggest that until the mid-1980s, these representations were dominated by notions of the “golden years” of retirement as a period of well-earned rest, followed by a notion of active, individualist, and consumerist aging in a silver economy, which, since the mid-1990s, has become contested by ideas cantering on productive aging. A 2009 campaign by the German Federal Ministry for Seniors called “Count deeds, not wrinkles” is illustrative of this discursive turn in productivity: Retirees are increasingly expected to give back to a society that pays their pensions, and they continue to work with or without pay (ibid.). Hence, the retirement transition may be normatively evaluated based on how active and productive everyday lives of transitional individuals are assessed to be.

If we now combine this sensitivity for normativity of retirement transitions with the theory of resonance, we can ask: How do chrononorms shape experiences of resonance in the transition process to retirement? How are individuals with non-normative retirement transitions confronted with “world-muting” and repulsive reactions from others and the world?

### Resonance and the Implications of Social Inequality in the Retirement Transition Process

Another and closely related aspect to consider regrading successful or unsuccessful retirement transitions are questions of loneliness and social exclusion as well as social inequality (De Jong Gierveld 1998; Walsh et al., 2017; Segel-Karpas et al., 2018).

Regarding gendered retiring, Kim and Moen (2002) state, that male retires are less satisfied with the income changes resulting from retirement than women, who in turn are more sensitive to relationship conflict and retirement coping. From the perspective of financial resources, women are more in the risk of lower financial resources in old age, due to shorter, interrupted working biographies and less possibilities for financial assets (Lusardi and Mitchell 2018). Additionally, for women living the traditional male-breadwinner model retirement, in the sense of leaving paid employment does not appear at all, it rather takes place at an early stage, when children leave home, and they “retire” from reproductive work. However, they traditionally do not “retire” from unpaid household duties.

Scharf et al. (2001) revealed how social exclusion during the retirement process emerges in multiple dimensions: in terms of participation and integration, spatial segregation and institutional disengagement. Burgess (1960, 20–1) uses the term “role-less role” to describe older persons in retirement as individuals who have been sidelined by society because of negative stereotypes of old age. Furthermore, persons with low socio-economic status, fragile social networks, singles, or individuals, who exit the labor force involuntarily are more likely to experience retirement transition as critical life-event and have less possibilities to establish successful strategies as retirees (Ebbinghaus and Radl 2015; Segel-Karpas et al., 2018; König et al., 2019). Limited socioeconomic resources when retiring also have moderating effects with regard to mental health impairments such as stress and depression, lower levels of locus of control, self-efficacy and sense of coherence (Antonovsky and Sagy 1990; Kim and Moen 2002; Vo et al., 2015; Hessel 2016; Topa and Valero 2017). Thus, on the one hand mental health status influences the timing and probability of giving up gainful employment and on the other hand, the retirement transition shapes indicators of wellbeing in old age, especially for subjective health, health behavior and feelings of social exclusion and loneliness.

If we now combine this sensitivity for the (re-)production of social inequalities in retirement transitions with the theory of resonance, we can ask: Do experiences of resonance in the retirement transition vary based on social position, and (how) does this experience contribute to the (re-)production of social inequalities?

## Methodological Approach: Mixed-Methods Research

In order to empirically discuss the issues raised above, we use a mixed-methods research design (MMR) and can thereby consider both qualitative and quantitative data. Unlike classical data triangulation, which aims at describing phenomena with data – as diverse as possible – to validate results and minimize measurement errors (Denzin 1978), our approach aims at multi-perspectivity, which is likely (and should) lead to complementary or contradictory results (Flick 2011). Our approach to mixing methods and data can be understood as reflexive-dialectical (Hesse-Biber and Johnson 2015), whereby a dialogue between the method (ologies) used is sought so that the reflexivity of our own research is strengthened.

### Quantitative Data From the German Ageing Survey

Quantitatively, we evaluate four waves (2008–17) of the nationwide representative German Ageing Survey (hereafter: DEAS), which evaluates the life situation of people in the second half of life (40+). Of all respondents who participated in at least two waves, 1.240 experience the transition into retirement during the observation period. In order to reflect the different facets of the transition, the analysis distinguishes between transitions to retirement from active employment (*n* = 632) and from a phase of non-employment (*n* = 497) as well as transitions from active or non-employment to a reduced-earning-capacity pension (*n* = 111).^3^

The operationalization is based on the core ideas of Rosa’s resonance concept, but with the awareness that the complexity of the overall construct “resonance” can only approximately be grasped. The resonance as well as dissonance relations are based on the Positive and Negative Affect Schedule (in short: PANAS, after (Watson et al. 1988; Engstler et al., 2019), that queries the frequency of certain affects along positive (e.g., enthusiastic) or negative (e.g., angry) adjectives and respectively summarizes them into an index, which is higher the more positive or negative affects one experiences. For the present analysis, the index for positive affect is used to operationalize the experience of resonance and the index that maps negative affect experiences is used to operationalize dissonance. As the constitutive flip side of resonance, the experience of alienation is measured by the frequency of depressive symptoms, which include physical experience (e.g., quality of sleep) as well as indifference (e.g., lack of motivation) and a repulsive sense of the self and the world (e.g., rejection by others).^4^ This operationalization is based on Rosa’s assumption that “(a) theoretical analysis of alienation (...) can simply take as its starting point the already confirmed increase in burnout- and depression-related illnesses” (Rosa 2019, 181). The relatively strong correlations of the scales for resonance, dissonance and alienation (R and D: 0.299 ***; D and A: 0.451 ***; R and A: 0.371 ***) already indicate their co-constitutive relationship (see Table 1).^5^ With operationalizing the frequency of negative affects as dissonance we aim to account for the productive possibility and openness of negative affects speaking to or touching an individual and leading to an answer, which can become a resonant or alienating experience – in opposition to nonproductive negative affects, which indifferently leave one untouched in the first place and result directly in alienating experiences. The dissonance scale displays the ambivalent in-betweens of resonance as a purely positive affective state – therefore the positive affects are operationalized as resonance – and alienation as a detached, indifferent state – which is indicated by depressive symptoms in our analysis. Here, we already see and will further discuss the challenge of empirically grasping the complex, ambivalent dimensions of resonance when operationalizing them.

**TABLE 1:** Correlation matrix (Pearson’s r).

	Resonance	Dissonance	Alienation	A	N	R	Female	Education	Age	Income	Partnered	Health, subjective	Health, functional
Resonance	1												
Dissonance	–0.299***	1											
Alienation	–0.372***	0.451***	1										
A	–0.107***	–0.124***	0.028***	1									
N	–0.001	–0.114***	–0.065***	–	1								
R	–0.100***	0.063***	0.186***	–	–	1							
Female	0.052***	0.126***	0.098***	–0.054***	–0.153***	–0.001	1						
Education	0.158***	–0.062***	–0.126***	–0.117***	0.053***	–0.085***	–0.200***	1					
Age	–0.120***	–0.151***	0.014*	0.830***	0.583***	0.087***	–0.081***	–0.100***	1				
Income	0.143***	–0.050***	–0.119***	–0.160***	0.045***	–0.087***	–0.051***	0.231***	–0.066***	1			
Partnered	0.076***	0.002	–0.138***	–0.133***	–0.037***	–0.079***	–0.187***	0.130***	–0.166***	0.116***	1		
Health, subjective	0.322***	–0.199***	–0.455***	–0.158***	–0.008	–0.253***	0.017**	0.129***	–0.150***	0.142***	0.075***	1	
Health, functional	0.279***	–0.125***	–0.399***	–0.307***	–0.079***	–0.372***	–0.080***	0.190***	–0.336***	0.129***	0.160***	0.558***	1

Notes: A = active employment versus old-age pension, N = non-employment versus old-age pension, R = active/ non-employment versus reduced-earnings capacity pension. Significant on a *5%-level, **1%-level ***0.1%-level; German Ageing Survey (DEAS)

Furthermore, we control for gender, education, chronological age, relationship status, net equivalent income, and subjective as well as functional health.^6^ Hybrid panel models (Rabe-Hesketh and Skrondal 2008) are used to examine inter-individual differences as well as intra-individual changes in the experience of resonance, dissonance and alienation during the transition to retirement. How different everyday practices, social relationships and loneliness^7^ promote as well as prevent resonance, dissonance and alienation experiences is analyzed based on descriptive correlation coefficients for individuals who are in the post-acquisition phase.^8^

### Qualitative Data From the “Doing Retiring” Project

Quantitative data is complemented with qualitative case studies from a qualitative longitudinal study, which is part of the project “Doing Retiring – The Social Practices of Transiting from Work to Retirement and the Distribution of Transitional Risks” at Goethe University Frankfurt (2017–21). The study follows 30 individuals between the employment and retirement over 3 years with episodic interviews according to Flick (2007), photo and activity diaries, and observations. Sampling was done through notices and display of flyers in public places (e.g., supermarkets), through businesses as well as social and recreational organizations (e.g., University of the Third Age), and through personal contacts. In this paper, we refer in particular to the episodic interviews collected during the first (2017–18) and second (2019) waves. The interviews began with a narrative-generating introduction, which in the first survey wave was biographically oriented and in the second focused on what had happened in the lives of the participants during the previous year. This introductory narrative was followed by an inquiry section with more specific questions that focused on the experience of the transition and the organization of everyday life in the employment and retirement. The interviews lasted between one and 3 hours. They were fully transcribed and coded using MAXQDA analysis software. The coding procedure was based on the social constructivist Grounded Theory (Charmaz 2006): In a first step, the transcripts were completely coded by initial coding; in a second step, central codes were identified by focused coding, and in a third step, these codes were related to each other in axial coding. In all three steps, a cross-case and comparative incident-by-incident approach was taken in order to highlight shared or divergent patterns of practice rather than individual trajectories.

For this paper, changes in resonance relations during the transition into retirement are highlighted on the basis of three non-linear but distinct transition processes that deviate from the chrononorms of institutionalized life courses: one transition from full-time employment to unemployment to reduced-earning-capacity pension, one transition from full-time employment to indefinite leave of absence, and one transition from long-term unemployment to reduced-earning-capacity pension. These three trajectories were chosen to highlight the complexities, nonlinearities, and fractures of transition processes that cannot be mapped in such depth from quantitative data. Moreover, transitions that deviate from norms, which could be discursively framed as “failed”, are particularly suited to better understand those very norms and normalities.

The three case studies can be summarized as follows:

1. Richard,^9^ born in 1954, is married, has no children, and lives together with his wife in a terraced housing estate on the outskirts of a large Western German city. He lost his job in his late 50s, after working as an unskilled laborer in a factory for many years, which severely affected his health. After 2 years of unemployment, he has recently started receiving a reduced-earning-capacity pension at the time of the first interview.
2. Mia, born in 1955, is married with no children and lives with her husband in a large city in Western Germany. She has a degree and holds a management position in a large, international organization. At the time of the first interview, she has been on leave of absence for about 5 years due to burnout.
3. Jan, born in 1965, is married with no children and lives in a small town in Western Germany with his wife. He has had a frail employment history and most recently worked as part of the security staff at a hospital. At the time of the first interview, he has been unemployed for more than 5 years, works in job creation measures, and also receives a reduced-earning-capacity pension due to mental illness, including severe depression.

## Results

### Transformations of Resonance in Normative Transitions

The transition into old-age pension is being understood to be a normative transition into retirement. It can nevertheless make a difference if one retires from the status of being actively employed or non-employed. On average, these two groups show similar levels of resonance (active employment: 3.66; non-employment; 3.56) and dissonance (active employment: 2.06; non-employment; 2.06) prior to the transition to the retirement. However, individuals undergoing the transition from active employment to retirement show a lower average alienation (5.64) experience than individuals making the transition from non-employment (6.21).

For individuals who complete the transition into old-age pension from active employment during the observation period, resonant experiences become more frequent and dissonant as well as alienating experiences decrease significantly (see Table 2). With respect to the transition into old-age pension from non-employment, there is also an increase in resonant experiences and a decrease in dissonant experiences, although the alienation experience remains unchanged. These results retain their validity even when controlling for social differentiations – such as chronological age or functional health. Except for the ones transitioning from non-employment, where the effect of the experience of dissonance disappears. On the basis of these intra-individual developments, we can assume that the transition to old-age pension, opens up spaces – at least in the short term – that promote the emergence of resonance, the reduction of dissonance and alienation.

**TABLE 2:** Results of the hybrid panel regression regarding transformations of resonance, dissonance and alienation.

Transition…	…from active employment into old age pension			…from non-employment into old age pension			…from active or non-employment into reduced-earnings capacity pension		
	Resonance	Dissonance	Alienation	Resonance	Dissonance	Alienation	Resonance	Dissonance	Alienation
Between									
Employment-phase	–0.141***	–0.144***	0.488***	–0.001	–0.223***	–1.629***	–0.271***	0.160***	5.621***
									
Employment-phase	0.067**	–0.035	–0.193	0.115***	–0.073**	–0.898***	0.019	–0.019	0.981**
Female	0.088***	0.107***	0.603***	0.100***	0.099***	0.655***	0.655***	0.135***	1.073***
Education	0.082***	–0.011	–0.163*	0.089***	–0.014	–0.239*	0.095***	–0.008	–0.189
Age, chronological	–0.004***	–0.008***	–0.056***	–0.005***	–0.008***	–0.053***	0.000***	–0.010***	–0.065***
Income	0.000***	–0.000*	–0.000***	0.000***	–0.000	–0.000**	0.000***	0.000	–0.000***
Partnered	0.037**	0.050***	–1.045***	0.054**	0.050**	–1.104***	0.045*	0.011	–1.269***
Health, subjective	0.176***	–0.153***	–2.562***	0.171***	–0.142***	–2.849***	0.200***	–0.178***	–2.886***
Health, functional	0.003***	–0.001**	–0.058***	0.003***	–0.000***	–0.054***	0.003***	–0.001*	–0.052***
Within									
Employment-phase	0.051**	–0.081***	–0.690***	0.046*	–0.036*	0.062	–0.015	0.004	0.275
									
Employment-phase	0.070***	–0.091***	–1.064***	0.068**	–0.034	–0.133	–0.005	–0.008	–0.501
Age, chronological	–0.000	–0.002	0.010	0.003*	–0.000	0.021	0.006***	–0.004**	–0.035*
Income	0.000	0.000	0.000	0.000	0.000	0.000	0.000	–0.000	–0.000
Partnered	0.019	–0.021	–1.369***	0.006	–0.007	–1.378***	0.019	–0.050	–1.879***
Health, subjective	0.057***	–0.041***	–1.569***	0.056***	–0.028***	–1.523***	0.083***	–0.070***	–1.773***
Health, functional	0.003***	–0.001***	–0.050***	0.002***	–0.001***	–0.049***	0.003***	–0.002***	–0.062***

Notes: Significant on a *5%-level, **1%-level ***0.1%-level; German Ageing Survey (DEAS).

Regarding differences between groups, we find, that old-age pensioners do experience less resonance and dissonance as well as more alienation than actively employed individuals. But when controlling for social differentiations, the effect for dissonance and alienation become insignificant, leading to the assumption, that other factors of social differentiation are responsible for the univariately observed differences. The effect of resonance remains significant, but changes its direction, meaning that with controlling for other systematic differences, individuals in active employment do experience less resonance that those in old-age pension. Comparing the non-employed and old-age pensioned, the former seem to experience more dissonance and alienation – also when controlling for social differentiation. In the univariate models no influence of employment status on resonance is found, but with the control variables, it becomes apparent, that old-age pensioners do experience less resonance that the non-employed.

The transition into retirement can also be accompanied by other transitions or processes, which also reflect intra-individual transformations of dimensions of social inequalities. With regard to these parallel processes, it becomes apparent that an improvement in subjective or functional health promotes the experience of resonance and, at the same time, considerably weakens the experience of dissonance and alienation, irrespective of the employment situation prior to the transition.^10^ In contrast, if a person becomes single during the same period, the experience of alienation is significantly strengthened, even though the experience of resonance and dissonance does not change. Increases in chronological age do not result in resonance-enhancing, dissonance-promoting or alienation-dynamizing moments for individuals transitioning out of active employment. The same holds for individuals who retire from non-employment, except their experience of resonance is becoming lower, the older they get. A change in income – in the regarded transition usually a reduction – does not alter resonance, dissonance, and alienation experiences. In summary, the analysis of the normative transition into old-age pension reveals transformations of resonance, dissonance, and alienation experiences, which, however, do not result solely from the transition itself, but rather from a mixture of parallel transitions (e.g., into singlehood) or processes (e.g., ageing).

As any other transition, the transition into retirement does emerge in the realm of social inequalities. With between estimators we can observe, if there are systematic advantages or disadvantages between social groups in experiencing resonance, dissonance, or alienation. Similar to the intra-individual changes, subjectively as well as functionally healthier individuals mostly tend to experience more resonance and less dissonance as well as alienation than those who are less healthy. Gender and age specific observations indicate that women and younger individuals compared to men and older individuals have a relationship to the world, which is more pronounced in general, meaning, that is at the same time more resonant, dissonant, and alienating. With regard to education we see, that the higher educated do not only experience more resonance, but also more less alienation, while dissonance does not vary with education. Comparing partnered and single individuals, we see, that the latter show lower degrees of resonance and dissonance, but higher degrees of alienation. Since these results are – in contrast to the intra-individual results – significant, we can assume that a relationship transition does affect resonance relations not in the short –, but in the long-term. We do not find any differences between income groups, which is in line with the finding, that also changes in individual income do not affect resonance, dissonance, or alienation. These inter-individual results are mostly the same for both normative transitions.

### Transformations of Resonance in Non-Normative Transitions

The transition from active or non-employment to reduced-earning-capacity pension is understood to be a non-normative transition into retirement. For this transition, active and non-active employees are combined, even though – as we saw in 5.1 – the pre-transitional situation can make a considerable difference, but the small number of cases does not allow for further differentiation. Comparing the levels of resonance (3.31), dissonance (2.34) and alienation (11.78) of the non-normative transition to those of the normative transitions, we see, that the resonance level of the former is lower than that of the latter. The dissonance level is slightly and the alienation level is considerably higher for the non-normative transition (*Transformations of Resonance in Normative Transitions*).

Our results illustrate, how completing this transition does not affect any of the three dimensions of resonance, as the corresponding coefficients are not significant – not even when controlling for social differentiations (see Table 2). Therefore, the transition to a reduced-earning-capacity pension is, neither resonance-enhancing nor dissonance- or alienation-dynamizing; rather, no resonance transformation accompanies this transition.^11^

Regarding parallel transitions and processes, we find similar patterns as we found for normative transitions. It is nevertheless remarkable, that increasing age raises resonance and decreases dissonance as well as alienation in the transition to reduced-earning-capacity pension, because ageing does not influence resonance relations in the normative transition from active employment. Age specific differences do however not affect resonance, meaning, that the younger are not more or less resonant within the world than the older. This difference in inter- and intra-individual effects of the age points to the importance of how retirement is framed normatively, since age does not influence normative, but non-normative transitions. We find another notable difference in the observation, that – when regarding the transition into reduced-earning capacity pension – there are no differences between educational groups regarding alienation. The other group specific differences affect resonance, dissonance, and alienation in nearly the same way as they do in normative transitions.

How experiences of resonance transform (and are transformative) in the practical process of “doing transitions”, however, remains invisible in the quantitative analysis. Looking at the resonance trajectories in the selected case studies, dissonances and/or losses of resonance at the end of working life are evident all three selected cases. The loss of resonance is often experienced and narrated through bodily affects by the research participants. Both Richard and Mia describe feeling “drained” and having “no energy left.” They also observe a decreasing interest in their work and an increasing affective distancing. Richard describes this process as an “inner emigration”, which he tells it as follows:

“I still did my job well, I think, but, how shall I say, I didn’t paint the town red anymore. I did what was necessary, what could be achieved, and I did that all steady. It didn’t bother me as much anymore, the anger that had previously cost me a lot of nerves with the management. I always took it very much to heart. And took a lot of it personally. [...] the drive was missing in the end. It was no longer, “Yes, hurrah, we’ll fight, we’ll do, we’ll do, yes!”, but it was, “Yes, I’ll do it.” And I think that happens to every person who is already inwardly saying goodbye, to the profession.”

While the process of affective disengagement, as Richard describes it, is gradual for some, others refer to specific triggers, often characterized by a lack of recognition, a reduction in skills, autonomy, or respect. Mia talks about the moment when her autonomy was curtailed by a new supervisor and she retrospectively “very likely should have made the break”:

“And then he said to me, you don’t decide here, we decide when and where this takes place. And that was the first gut punch.”

What is being experienced as recognition differs according to setting and social position. While it is painful for Mia as a project manager to be partially deprived of decision-making authority, Jan as a security guard emphasizes simple practices, such as being greeted, which shows him that he is being seen in his professional life and that something – or in this case, specifically, someone – speaks to him:

“Good morning, Mr. Meier, have a nice weekend, Mr. Meier, (takes a breath). Where are you going today, well, have fun!’ That does something, like for the psyche.”

What is striking in all three cases is that the experienced transformations of resonance on their transitional paths are relatively similar, despite the different social situations and positions they depart from: Mia, the highly educated manager in an international organization, finds herself experiencing similar dissonance and alienation as Richard, the unskilled worker, and Jan in his precarious employment situation.

However, looking at the further development of experiences of resonance after the end of working life reveals major differences between the cases – which seem, however, again unrelated to socio-structural inequalities: While Richard very quickly finds a new sphere for experiencing resonance in care activities for others, Mia and Jan find themselves in a largely resonance-less but searching “state of uncertainty” for a longer time, which Mia describes as follows:

“Sometimes I feel really good, full of energy, trying to take care of myself. Then sometimes I’m so full of energy that I think, I can still pull something off. (begins to cry; in a drained voice) So sometimes I think like that, I would just say lost in transition. That’s kind of my state.”

However, the fact that experiences of resonance are processual and changeable can be seen both in biographical narratives and also very clearly in the longitudinal monitoring of the study participants. At different points in the transition process, the interviewees report different experiences of resonance, dissonance, and alienation, and people who lack experiences of resonance at one point in time may find them at a later point in time. Thus, lack of resonance is not a “fate” but arises in the context of relationships as well as in the execution of social practices and is thus changeable in social practices. This is exemplified in Jan’s narratives: while in the first interview he still describes his situation as “cruel”, “hopeless”, and “lonely”, he has found a new source of resonance a year later. Together with his wife, he moves into a communal living project, where he feels comfortable and has arrived, “[...] because they all have the same goal: We want to grow older together, as confidants [...] to share life”.

### The Role of Everyday Practices in Transformations of Resonance

The participants in the qualitative study often experienced the first phase after the end of working life as a quest, which, from a resonance perspective, can be understood as a search for new axes of resonance. This search is not purely intrinsically motivated, but is also societally, hence discursively and normatively, shaped – the study participants, for example, emphasize that they “must” look for new tasks, activities or projects, otherwise they are afraid to fall into inactivity, listlessness and/or depression, which is sometimes captured in the discourse of the “retirement hole”. Jan describes this experience as follows:

“Now (A.N. after the end of working life) everything is threatened to go down the drain, all my competencies will dry up then, that’s my feeling about it – and I do want to create.”

Mia is also looking for such a creative task. She says about herself, “Actually, I’m looking for a project.” But on the other hand, she is afraid of the obligations creative opportunities always comprise:

“On the other hand, I’m incredibly afraid that I’ll fall back into this rat race very quickly. I just don’t want that.”

Mia and Jan participate in a range of activities, which promise to enhance resonant relations: Jan talks about “individual therapy, psychosomatic clinics, pilgrimage, meditation, body therapy, choir singing – so you’ve got plenty of options there already” and, after losing his job, he goes directly to a monastery for 6 months. Mia says she, “does more sports, does an Ayurveda treatment, does yoga (…) does mindfulness exercises”, has “hired a woman to coach (her) because (she) was just in such a bad place”, and attends seminars on spirituality to “really engage more with (her)self”. She also tells about singing in a choir:

“(I) learned new things. I started singing, something I had totally neglected. So, making music, I’m doing that really intensively, being in the choir, taking singing lessons, that kind of thing. (…) I’ve learned a lot about my own psyche, how that works, about feelings.”

However, they both do not find any sustainable experiences of resonance in these activities. Richard, on the contrary, describes himself as having “really arrived” in retirement shortly after the end of his working life. He has tried similar activities as Jan and Mia but has finally arrived at “shopping, doing laundry, writing poetry”, which fills and fulfills his day. He experiences retirement as a phase of slowing down and turning towards the world, which enables him in the first place to reestablish meaningful self-world relationships. Richard recalls:

“I thought at the beginning I’d fall into a big hole or something and really need to find new kinds of work. But I didn’t fall into a hole, I went for a walk more often than usual. I enjoyed it.”

Recognition for what he does remains important to him. He tells about writing poetry, “And I enjoy it, especially when other people like it too and say, ‘That’s really nice!’” He also places emphasis on the importance of “experiencing new things”, reflecting on one’s own biography, and doing things that were not possible (in terms of time or structure) in the past. Richard now dreams of studying in order to “learn to understand people further.” In terms of the resonance concept, we similarly see a striving for expanding reach of the world: Richard not only wants to write poems, but he also wants other people to read and like them. But when asked about recognition, he responds that all he wants is respect – “not being cautious or anything, but just the normal respect that everyone deserves.”

While the qualitative findings provide a detailed insight into the specific transition practices and their meanings for those who are transitioning, we can determine at the quantitative level how specific everyday practices correlate with the experience of resonance, dissonance or alienation, when one is in the phase of retirement. To analyze which everyday practices foster resonance relations, correlation tables^12^ (see Table 3) – separated by the form of retirement: either old-age or reduced-earnings capacity pension – were calculated to indicate the relationship between the frequency of performing a practice and the intensity of the resonance dimensions. The results illustrate that the emergence of dissonant experiences does not relate to certain everyday practices, since the correlation coefficients are mostly non-significant as well as low in general and especially in comparison to those of resonant and alienating experiences.

**TABLE 3:** Correlation matrix for all resonance dimensions and everyday practices (Pearson’s r).

	Resonance		Dissonance		Alienation	
	O	R	O	R	O	R
Everyday						
Church attendance (also mosque, synagogue)	–0.007	0.038	0.070***	0.018	–0.029*	–0.043
Going for a walk	0.071***	0.111*	–0.010	–0.095	–0.081***	–0.084*
Crossword puzzles/Mental exercise	0.069***	0.177***	–0.019*	–0.080	–0.053***	–0.077
Attendance: sports events	0.073***	0.029	0.009	–0.055	–0.073***	–0.096*
Artistic activities	0.112***	0.053	0.013	0.075	–0.056***	0.020
Gardening (in the summertime)	0.116***	0.067	–0.013	–0.092*	–0.145***	–0.162***
Handicraft and DIY work	0.143***	0.099*	–0.044***	–0.062	–0.109***	–0.105**
Attendance: courses and lectures	0.133***	0.214***	0.025**	–0.046	–0.068***	–0.134**
Attendance: Political events	0.156***	0.158***	–0.013	–0.048	–0.082***	–0.137***
Using a computer	0.020***	0.114**	–0.030**	–0.006	–0.114***	–0.064
Sports	0.184***	0.163***	0.009	–0.031	–0.155***	–0.144***
Attendance: Cultural events	0.228***	0.195***	–0.009	–0.010	–0.182***	–0.133**
Housework	0.068***	0.111*	0.026**	0.013	–0.042***	–0.018
Social relationships						
Contact to own children	0.008	0.065	0.008	–0.055	–0.011	–0.060
Contact own grandchildren	0.039**	0.200	–0.006	–0.210	–0.056***	–0.009
Contact own parents	–0.001	0.129	0.026	–0.083	–0.030	0.000
Board or parlor games	0.079***	0.072	–0.019*	–0.000	–0.070***	–0.042
Attendance: senior groups	0.030	0.031	0.014	–0.090	–0.010	–0.070
Attendance: other groups	0.077***	0.038	0.021	–0.030	–0.073***	–0.057
Meeting with a steady group	0.114***	0.182***	–0.014	–0.091*	–0.106***	–0.150***
Visits from friends	0.146***	0.149***	–0.031**	–0.080	–0.115***	–0.164***
Loneliness	–0.357***	–0.436***	0.422***	0.533***	0.296***	0.389***

Notes: O = Old-age pension, R = Reduced-earnings capacity pension; Significant on a * 5%-level, ** 1%-level *** 0.1%-level; German Ageing Survey (DEAS)

The formation of resonant relationships in everyday practices, on the other hand, seems to be particularly enhancing for old-age pensioners by attending (cultural or political) events and courses, by using the computer in their free time, by body-related practices such as gardening or sports, by creative practices such as artistic activities or handicraft and DIY work. Most of the correlations between resonance and everyday practices are also found among pensioners with reduced-earning-capacity pension: For them creative practices and gardening do not promote resonance, whereas walking, housework, and crossword puzzles or mental exercises are found to be resonance-enhancing.

The extent to which alienation and resonance are interconnected is suggested by the similarity in everyday practices favoring them: the experience of alienation is attenuated by similar everyday practices as the experience of resonance is being enhanced. More precisely, handicrafts, gardening, sports, and attending cultural events are equally alienation-reducing, no matter which form of retirement. Differences between old-age and reduced-earning capacity pension show that the alienation experience of the former is reduced using the computer for leisure activities and of the latter by attending courses or lectures or political events.

The everyday practices promoting resonance can be assigned to different resonance axes: politics, art and nature, self-effective work, sports and consumption (Rosa 2019). It is particularly striking that practices such as church attendance – contrary to the theory-based expectation that religion can be a resonance-enhancing axis – seem to have a vanishingly low correlation with the three dimensions of resonance. We see however in the qualitative data, that spiritual or transcendent practices, like pilgrimage, meditation, or Ayurveda, are – at least an attempt – to experience resonance, which suggests, that maybe resonance and traditional or institutionalized religiousness are not closely tied to each other.

At the same time, none of the everyday practices turned out to be alienation-dynamizing, which could be related to the operationalization of alienation with depressive symptomatology and the association of high depressive symptomatology with a less active everyday life.

### The Role of Social Relationships in Transformations of Resonance

Social relationships constitute a crucial axis of resonance across the life course, and life course research has pointed to the relevance of “linked lives” (Elder et al., 2003) in the study of transitions. In particular, Mia and Jan lack a sense of community in their search for resonance. In this regard, both describe a sense of distancing from others that can be captured as alienation. As Jan puts it, “I think they’re all somewhere completely different than where I am – we’re all quite distant.” He speaks much of his urgent desire “to belong, to have a group, a gang,” and attributes the lack of such to the fact,

“(…) that I’m retired so early because most men don’t show up, or there aren’t any, or they don’t federalize. Most go the natural way and retire early at 60 (takes a breath) and there from is the way of division (…) and then I have to see here just as a man (takes a breath), early retiree, 52, where I stay (takes a breath) and that is already difficult, to be perceived and understood with like-minded people, that is incredibly (takes a breath) hopeless.”

He nevertheless finds some kind of resonant relation with his new house mates in the collaborative living project who, unlike him, are not experiencing retirement transitions, but are just within very different transitions in the life course, e.g., starting a family or moving in together as a young couple.

“They are enthusiastic young mothers, young fathers with 30, who simply want to create something. Just like me. I want to create something. For myself, my body, and with others and um with the housing project. Just in a different way with all of my experience.”

Richard finds resonance in supporting his wife more than in searching and finding resonance in the form of a new job or an exploration of his self. Asked about changes in his marriage accompanied by his transition out of the workforce, he thus replies:

“We treat each other even more appreciatively (…) since I retired and have more time to get things done on the side (…). So, I have the feeling that it has become even warmer than it already was. That is a positive side effect. Yes, I don’t know what it is, but (takes a short breath) maybe because I have time to take care of things. (…) You didn’t have that before at work, it was so stressful or you were stressed and didn’t have time (…) instead to enjoy this free time COMPLETELY consciously. So that’s really becoming aware of what kind of time you have at the moment, that’s – I can’t say it often enough, how happy I am.”

In addition to caring for his wife, Richard, who is childless himself, also builds new intergenerational relationships with other children and young people in transition, serving as a mentor for refugee minors and as a read-aloud grandpa. He thus feels as a part of a social community and has the feeling that he “has both feet on the ground” much more than he did during his working life.

The quantitative analysis of social relationships and loneliness is in line with the qualitative finding of searching for a community. As we already saw for the everyday practices, dissonance is also not attenuated by social relationships (see Table 3). Regarding resonance and alienation, meeting with friends or a fixed circle of people enhances resonance as well as reduces alienation for both forms of retirement. From a resonance-theoretical point of view, the family functions as a “harbor of resonance” (Rosa 2019, 341, 435f.). It is therefore rather surprising, that having contact to any family members is *not* tied to resonance transformations. Feeling lonely and experiencing resonance, dissonance, or alienation is interrelated as follows: the lonelier one feels, the lower the experienced resonance and the higher are dissonant and alienating experiences. We additionally see that the correlations of loneliness with resonance, dissonance, and alienation are stronger for reduced-earning capacity than for old-age pensioners, which strengthens our qualitative result, that finding (or not finding) a community is especially pivotal within non-normative transitions.

## Discussion

In this paper, we posed the question of whether, and if so, how, resonance experiences according to Rosa are transforming in retirement transitions. At the backdrop of our empirical analyses, we want to summarize the main results:

First, persons in transition to retirement seem particularly sensitive for unpredictable experiences of resonance and alienation characterized by a balance of fears and hopes for the future. Secondary data analysis of the DEAS suggests that the retirement transition *opens spaces of resonance* for many people, while at the same time spaces of radical dissonance and alienation – most often caused by work or the reconciliation of work and family/leisure – are closing. The qualitative case studies enrich this finding by suggesting a loss of resonance at the end of working life – illustrated, for example, in phenomena such as “internal resignation”. This suggests a certain phase structure of resonance experiences or a “resonance choreography” throughout the retirement transition, namely a tendency toward losses of resonance at the end of the employment phase, followed by a gain in resonance in the early stages of retirement. This finding is both in line with studies that have hinted at different phases in the retirement transition like Turners (1969) differentiation of a separation, a liminal and an incorporation phase, where the strive for Communitas may represent desire for horizontal resonance axes. Combining Atchley (1971) phase model with resonance theory, we could interpret the pre-retirement phase as mode of desire for resonance, whereas “in the honeymoon phase” and “phase of reorientation” spheres and axes of resonance are explored and possibly established. However, it remains open how and for whom such spaces of resonance emerge at the transition from work to retirement.

Second, our quantitative results show – and thereby partly answer the question posed in the previous section – the importance of the timing of the retirement transition within the life course. Rosa (2019) understands such discursive constellation to be “moral roadmaps” (132) for experiences of resonance. The closer the transition happens around the statutory retirement age – the more “normal” or “right” it seems – the more resonance and the less dissonance and alienation do people experience in the course of it. This finding is in line with concepts like *chrononormativity* (Freeman 2010) as the norms around “right timings” in the transition from work to retirement (Wanka 2019a), and the institutional and discursive “transition regimes” (Walther 2020) that set such systems of chrononorms in place. Qualitative case studies increase our understanding of the importance of perceived normality for experiences of resonance even further: If a person retires at a similar age and in a similar life situation as others around them, they feel a connection to the world that is otherwise unavailable to them. Leaving, in contrast, the workforce well before statutory retirement age and thus “violating” not only transitional regimes but also the “chrononormative life course regime” (Kohli 2007; Freeman 2010) conditions experiences of dissonance and alienation rather than resonance, as we can see in the cases of Mia and Jan. This is due in significant part – and here we can again connect to anthropological transition research – to the absence of a community or “communitas” which experiences the transition together (Turner 1969).

*Third,* we find different resonance, dissonance and alienation transformations depending on the social position, specifically 1) whether a person transitions from active employment or non-employment to 2) old-age or reduced-earning-capacity pension, 3) when and in which life situation the transition occurs, 4) whether and how a person experiences recognition at the end of his or her working life, and 5) which opportunities to participate in resonant practices open up for him or her (or not). Even when our quantitative results show, that neither increases in income nor differences between income groups lead to more experiences of resonance. Disposition and spaces for resonance experiences are framed by contextual, sociostructural and cultural conditions such as connotations of masculinity and femininity or cohort-specific perceptions (Rosa 2019). In our data we found income-specific differences in resonance-enhancing and alienation-dynamizing everyday practices, e.g. going for a walk is for free, but for creative practices one needs to buy material. Thus, our results regarding health-, gender-, and education-specific differences are highly in line with known implications of these social inequalities, where subjects are differently able to experience, practice and express resonance.Whereas these three results are in line with existing concepts and results of aging, life course, and retirement research, the study also unveils two new pathways for further research: The *first* hints to a practice-theoretical expansion of the concept of resonance, the *second* to a relational expansion of retirement research.

1) Empirical results have shown that experiences of resonance do not just happen (or disappear) “naturally” in the transition to retirement but emerge from people’s engagement in *social practices.* Whereas Rosa assumes that the strive for resonant experiences and the aim to avoid alienation are basic human needs, our study shows the potential that lies in understanding resonance as *enacted* in social practices, and in asking which practices exactly open up spaces for experiences of resonance in the retirement transition, and for whom. As our results illustrate, this can be in ordinary everyday practices such as housework or taking a walk, as we see in Richard’s case. However, quantitative analysis also makes it apparent that there are no universal practices that create experiences of resonance equally for all transiting individuals – rather, such resonant practices depend on socio-cultural milieus.

As the qualitative case studies indicate, the phase directly before and after the end of working life is marked by a search for constellations of practices in axes in which experiences of resonance emerge. This search leads the study participants to a “market” of resonance-promising oases, that has established itself around the transition to retirement – ranging from senior traveling over adult education centers, from (psycho)therapy or counseling to volunteering, choirs, hiking associations and mindfulness workshops. Speaking with resonance theory, participating in such commercialized practices can be understood as attempts to purposefully appropriate the social world and intentionally create experiences of resonance. However, such “simulations of resonance” are often doomed to fail, since resonance is characterized by unavailability – it cannot be purposefully planned or (commercially) organized, as we see in the cases of Mia and Jan. Where people experience resonance is thus partly arbitrary. What seems to be central for experiencing resonance, however – following both the theoretical assumptions as well as the empirical material – is deceleration with retirement: Slowing down allows for adaptive world transformation and thereby transcending the self.

2) Our results explicate the relationality of the retirement transition (and life course transitions in general). Transitions, it becomes apparent, are linked between people – for example, the retiring person and their employer or partner – but also among themselves: We can see this relationality both quantitatively and qualitatively, since resonance transformations rarely arise from one transition alone, but from an *arrangement of cumulated and clustered transitions* occurring in sequence or parallelly, hence in temporal and interpersonal relation to each other, for example the transition to grandparenthood, partnership transitions, or relocation. In terms of resonance theory, the observation of a single transition falls short of identifying any transformations of the relationship between subject and world. Hence, it is not enough to think relationality in the life course only between different people’s lives, as in the life course research concept of “linked lives” (Elder et al., 2003), but also between different transitions occurring on one or more people’s life courses as “linked transitions”.

## Conclusion

Based on the results outlined above, we want to call attention to the importance of Rosas concept of resonance and its transformation in transition to retirement. As “the sphere of work is thus multiply charged with resonance” (Rosa 2019, 236), the final exit from the labor market represents a specific moment of individual porosity, in which unpredictable experiences of resonance, radial dissonance or even alienation may occur. The interaction between subject and segments of world is the result of social practices and capacities for resonance. We thus argue for a relational as well as practice-theoretical perspective on resonance transformations in the retirement transition (Wanka 2019b). The combination of these three theoretical and conceptual angles – resonance theory, practice theories and relational approaches – allows for more sociologically informed perspectives aside traditional models of push and pull factors on the retirement transition that can be fruitful for societal diagnoses of our times.

Finally, however, we would also like to address some criticisms of the resonance concept and limitations of this paper. First, some difficulties arise in measuring resonance, or rather our attempt to operationalize Rosa’s abstract resonance theory and thus make it fruitful for empirical retirement research. In our mixed-methods research design it becomes apparent that the operationalization and analysis of experiences of resonance differ between quantitative and qualitative data, each rendering different aspects of these experiences (in-)visible. The individual scales in the quantitative secondary data can only represent the complexity of the relationship to the world – if at all – in a fragmentary and thus highly abbreviated way. Moreover, it is hardly possible to adequately operationalize the ambivalence inherent to the concept – the dialectical relationship between resonance and alienation as well as their dissonant intermediate forms – with existing survey data, as we saw quite clearly in the results concerning dissonance, especially when analyzing everyday practices. Even though the detection of experiences of resonance seems easier in the qualitative data at first, the difficulty of translating, for example, bodily-affective experiences of resonance into linguistic articulations becomes apparent here. Interestingly, it is easier to verbalize aspects of alienation than experiences of resonance. Empirical research on resonance therefore arguably requires complementary other, non-linguistic methods, such as those used in ethnographic methodologies, visual procedures, and especially affect analyses. In this context, Robert Seyfert (2019) argues for an intensive sociology that – in contrast to an extensive sociology focused on social effects and causalities – is able to focus precisely on those processes. Altogether, we argue for systematic comparison and reflection of data generated with mixed and multi-method research to develop the concept of resonance further through its empirical investigation.

Second, even though Rosa hints towards questions of social inequalities – namely how the ability to experience resonance differs depending on one’s social position and how it changes across the life course, he in large parts avoids a detailed argumentation on the connection between resonance and social inequalities. This may in part be due to Rosa’s rejection of the resource-orientation of the sociology of social inequalities. But he himself does not get out of this logic either, when stating, that the educational system – and thus higher education – promotes higher resonance capacities for privileged social groups (Rosa 2019, 453). We see however based on our quantitative and qualitative results, that the experiences of resonance – and the attempt to appropriate it – are highly biased by one’s social position. For example, Jan and Mia try to appropriate resonance with consuming commercialized offers, and even though they partly fail to experience resonance, they can afford to try it.

Integrating the concept of resonance in the life course approach, we can on the one hand (re)negotiate successful aging, since resonance theory makes us sensitive to not solely regard the distribution of socio-economic resources but rather the distribution of resonance dispositions and capacities. With cumulative advantage/disadvantage theory (CAD; Dannefer 2003) we could argue that the ability to experience resonance depends on social status and cumulates – as other privileges – across the life course. As Rosa (2019) himself (albeit only marginally) mentions cultural gender or cohort-effects of resonance experiences, it could be fruitful, to further investigate how biographically and/or cohort-based inequalities and different life-course events and outcomes – ergo social stratification or the accumulation of opportunities and risks (Dannefer 2003) – relate to different dispositions and experiences of resonance. From this perspective we are able to evaluate resonance transformations without (re-)producing the detachedness of resonance theory and social inequalities ourselves.

Based on the results of the qualitative data, for example on everyday practices and the search movement for resonance, we can formulate further new questions for a theoretical and empirical approach to resonance: How, for example, is the social acceleration that Rosa proclaims related to a pluralization of transitions noted by reflexive transition research? Can we understand the design and ritualization of transitions as an expression of desire for resonance? What method (olog)ic approaches, such as ethnographic approaches, might help establish a “research architecture” related to resonance and transitions? If we operationalize resonance: does it represent a depended or independent variable and outcome for transition processes like retirement? How can we understand retirement decision-making and adjustment to retirement from the perspective of resonance theory? Which role plays voluntary of exit the labor force? How distinct is the theoretical differentiation between dissonant/resonant experiences and alienation? How can Rosas dialectical approach be linked to ambivalence or changing perceptions of resonance axes over time? Is it possible to state from a reflexive research perspective that resonance and the search for responsivity are basic human needs?

Concluding we would like to highlight, that resonance as a lifelong process offers the possibility to understand the transformational potential of transitions openly and in terms of a good life. It remains open, how resonance can be reflexively developed through its “empirical friction” on the phenomenon of transitions. The openness – e.g., towards temporal, material, and spatial dimensions of social life – inherent in resonance theory makes it appealing especially for reflexive transition research: A practice-theoretical and relational interpretation of the resonance concept in the sense of doing resonance by doing transitions, as we propose in this paper, opens up space – for resonant and dissonant discourses.

## Data Availability

The original contributions presented in the study are included in the article/supplementary material, further inquiries can be directed to the corresponding author.

## Footnotes

1 In his work Rosa generally draws on a variety of socio-philosophical and sociological traditions, such as Karl Marx’ early work, Erich Fromm, Theodor W. Adorno and Max Horkheimer from the Frankfurt School, Jürgen Habermas, Axel Honneth, Hannah Arendt, Bruno Latour, Charles Taylor, or Maurice Merleau-Ponty.

2 This term was developed by Eva Illouz (2018) to describe how commodities are being emotionalized: they are solely developed to produce affects and thereby promise certain experiences, emotions and in the understanding of this paper, resonance.

3 The respective dummy variables contain information on the pre-employment situation (0) and the form of retirement (1). To test the validity of this operationalization, fixed effects regressions were modeled with the duration of retirement as an independent variable. Once age and functional health are included in the model, duration no longer measures changes of resonance. Also, the chosen variable – as the rest of the data – allows to capture the transitions and the associated resonance transformations prospectively.

4 Short form (15 items) of the CES-D (Center for Epidemiologic Studies Depression) scale (Hautzinger and Bailer, 1993; Engstler et al., 2019).

5 The criterion validity of the resonance, dissonance, and alienation experiences was tested by correlating them with variables for self-assessment of distress (D) and change (C) due to the transition into retirement. The correlation coefficients (Pearson’s r) are low (resonance, D: −0.077***, C: −0.151***; dissonance, D: 0.166***, C: 0.085***; alienation, D: 0.198***, C: 0.203***) but operate in the same direction. Overall, the variables selected to measure resonance, dissonance and alienation appear to be quite independent of the self-assessment of the transition to retirement. Nevertheless, the variables for self-assessment itself are not as strongly correlated with each other as we expected (0.205 ***).

6 The variable gender is coded binarily (0=male, 1=female) and education is categorized in three levels according to ISCED. Chronological age is included in the analysis in years, and the variable is metrically scaled. The dichotomous, nominally scaled variable on relationship status maps whether respondents are single (0) or living in a relationship (1). Monthly equivalized income is a metrically scaled variable that reflects the economic situation. It is based on a construct variable – need-weighted on the basis of the OECD equivalence scale – that contains the respective monthly income of all members of a household over the age of 15 (see Engstler et al., 2019). The ordinal variable for subjective health assessment was recoded and has the following expressions: 0 = very poor, 1 = poor, 2 = average, 3 = good, 4 = very good. For functional health, we relied on the metric variable that transforms physical functioning in ten activities based on SF-36 subscale (Bullinger and Kirchberger, 1998; Engstler et al., 2019) into the standard 100 score. The higher the scores on this scale indicate a better the functional health.

7 The scale measuring loneliness contains the means of six items and is a short version of the loneliness scale from De Jong Gierveld and Van Tilburg (2006), see also Engstler et al. (2019), within which higher values indicate higher degrees of loneliness. Additionally, a measure to indicate social exclusion would have been interesting, from a theoretical point of view (*Resonance and Normativity in the Retirement Transition Process*), but indicators concerning this concept were only conducted in 2014 and 2017.

8 The DEAS allows the measurement of the frequency of different everyday practices, which are listed in Table 3. The respective variables were standardized and coded as follows: 0 = Never, 1 = Rarely, 2 = 1 to 3 times a month/ Several times a year, 3 = 1 time a week, 4 = Several times a week, 5 = Daily.

9 All data that allow conclusions to be drawn about individuals have been anonymized. All names used are pseudonyms.

10 We are concerned with an improvement in the individual’s health status or health assessment, i.e., resonance improves regardless of how healthy a person is in relation to others. In comparison, however, a change in subjective health assessment has a significantly stronger effect on resonance relations, which could be related to the observation that subjective health has a stronger connection with the dimensions of resonance relations than functional health (see Table 1).

11 The chosen evaluation method only allows to analyze changing and no constant resonance relations, therefore this result does not mean that no resonance is experienced during transition 3, but only that the transition itself does not affect the (existing) resonance relations.

12 On the basis of these correlation coefficients, it is not possible to draw conclusions about causal relationships, but they do reveal tendencies in terms of how everyday practices and resonance, dissonance and alienation relate to one another. Also only those respondents who were receiving either reduced-earning-capacity pensions or old-age pensions at the respective time of the survey were considered.
